# A Robust Feature Extraction Model for Human Activity Characterization Using 3-Axis Accelerometer and Gyroscope Data

**DOI:** 10.3390/s20236990

**Published:** 2020-12-07

**Authors:** Rasel Ahmed Bhuiyan, Nadeem Ahmed, Md Amiruzzaman, Md Rashedul Islam

**Affiliations:** 1 Department of Computer Science and Engineering, Uttara University, Dhaka 1230, Bangladesh; rasel.cse@uttarauniversity.edu.bd; 2 Centre for Higher Studies and Research, Bangladesh University of Professionals, Mirpur Cantonment, Dhaka 1216, Bangladesh; nadeem@uap-bd.edu; 3 College of Aeronautics and Engineering, Kent State University, Kent, OH 44240, USA; 4 Department of Computer Science and Engineering, University of Asia Pacific, Dhaka 1205, Bangladesh

**Keywords:** human activity recognition (HAR), feature extraction, feature reduction, enveloped power spectrum (EPS), linear discriminant analysis (LDA), multi-class support vector machine (MCSVM)

## Abstract

Human Activity Recognition (HAR) using embedded sensors in smartphones and smartwatch has gained popularity in extensive applications in health care monitoring of elderly people, security purpose, robotics, monitoring employees in the industry, and others. However, human behavior analysis using the accelerometer and gyroscope data are typically grounded on supervised classification techniques, where models are showing sub-optimal performance for qualitative and quantitative features. Considering this factor, this paper proposes an efficient and reduce dimension feature extraction model for human activity recognition. In this feature extraction technique, the Enveloped Power Spectrum (EPS) is used for extracting impulse components of the signal using frequency domain analysis which is more robust and noise insensitive. The Linear Discriminant Analysis (LDA) is used as dimensionality reduction procedure to extract the minimum number of discriminant features from envelop spectrum for human activity recognition (HAR). The extracted features are used for human activity recognition using Multi-class Support Vector Machine (MCSVM). The proposed model was evaluated by using two benchmark datasets, i.e., the UCI-HAR and DU-MD datasets. This model is compared with other state-of-the-art methods and the model is outperformed.

## 1. Introduction

Human activity recognition has (HAR) become a fascinating research area for researchers in the field of ubiquitous computing, and human-computer interaction because of its important contribution to monitoring daily human life activities [1,2,3]. It plays a crucial role in a wide range of real-life applications such as healthcare, elder care, smart home, fitness tracking, sports tracking, robotics, social security, industry, and so on [4,5,6,7,8,9,10]. Previously, contextual information played a big role in activity recognition [11]. This contextual information can be captured by placing sensors including cameras in the environment for activity recognition [12,13]. However, the experiment was conducted on a closed and controlled environment rather than a real daily life activity. Another popular approach is the use of body-worn sensors. This has the advantage that it can be used in both uncontrolled indoor and outdoor environments. Sensors are placed in different places of a body such as chest, wrist and ankle [14,15] to collect posture information. This method is less user friendly due to the fact that these sensors might cause inconvenience to the users. Moreover, data collection cannot be done surreptitiously in this approach. However, the aforesaid difficulties might be resolved in HAR applications by using smartphone sensors. Sensor embedded smartphones have become a cardinal part of our day to day lives over the last decade. Generally, these gadgets are embedded with versatile sensors such as accelerometers, gyroscopes, GPS, and magnetometers. The data is collected from the embedded sensors while the user performs any kind of daily activity including sitting, standing, walking etc. as long as users carry their phones with them. Thus, smartphone sensors can accumulate date without any additional hardware [16]. Accordingly, this approach for human activity recognition is appropriate for analyzing user’s daily activities continuously. Among all other inertial sensors, gyroscope and accelerometer are of our interest as human activities can be recorded via these sensors which are embedded in smartphones [17]. Activity recognition processed by the following consecutive steps including segmentation of data, feature extraction, selection of feature and classifier training [16]. Out of all the phases mentioned above, extraction of feature is the prime step because of its impact on the accuracy of classifiers [18]. Extraction of feature from sensory data has always been a challenging task due to the facts such as variable orientation of smartphones, placement and subject, unification of simple activities leading to a complex activity, the characteristics of smartphone sensor signals for the same activity will be different, which will degrade the recognition accuracy to a great extent [2,6]. One approach is to use hand-crafted features such as spectral entropy, energy of different frequency bands, auto-regressive and FFT coefficients which are used in much researche [19,20,21]. Despite of good performance in practice, domain-specific knowledge and generalization are required [22]. Thus, to surmount these shortcomings, deep learning-based models have been used in HAR systems to make the feature extraction process automatic [1,23,24,25]. Moreover, deep learning model does not totally require manual feature engineering as it is designed to train in an end-to-end fashion. In addition to high accuracy and good generalization, one main advantage of this approach is that after a deep learning model is created, it is trained in an end-to-end fashion which completely removes the need for manual feature engineering [18,26,27]. However, the computational cost required for this approach is infeasible for sensor-based phones with inadequate memory, processing and battery power [4]. Also, deep learning based techniques need a large number of annotated samples. In future, MobileNets-like systems will make this feasible which is an efficient deep learning models for mobile devices [28].

This paper has the target of a low-cost solution model that does not require domain specific knowledge and is able to extract quality features automatically. The EPS and LDA are used for feature extraction based on dimensionality reduction of signal spectrum. Finally, multiclass SVM is used with a view to classify the HAR. Later, the research is arranged in a following manner: Section 2 reviews the related literature; Section 3 represents the proposed scheme of activity recognition in detail; in Section 4, different experiments are performed to evaluate the performance of the proposed method; Section 5 concludes the proposed model.

## 2. Literature Review

Extract discriminative feature from raw signal is the key for HAR. For these reasons, useful features were extracted using Coordinate Transformation and Principal Component Analysis (CT-PCA) [6]. Centroid signature and histogram of gradient-based two Fourier descriptors are used in the raw signals to extract features [5]. Lastly, SVM and KNN are used as classifiers. The overall process had four sections. First, input signals as raw data, second, extraction of supplementary signal, third, extraction of feature, fourth, information fusion and last, classification. The average accuracy of feature level fusion SVM was 97.12% and K-NN was 91.75% and score level fusion SVM was 96.44% and K-NN was 84.02%. In different classification models using vision, particularly in image processing, the researchers proved that the variation of support vector machines has a greater capability to recognize the activities of video surveillance objects [29]. The researchers smartly used a mechanism that reconstructed the 2-D data so that it could able to interpret the inner meanings of movable objects. Though this research showed a good outcome of different physical activities but the major drawback was that it would not provide a workable solution in a real-life need. To tenacity the issue, in [30], the researchers used a group-based context-aware approach. Saha et al. implemented a statistical model and extracted a few statistical features from the raw signals [31]. SVM, KNN, and EoC are used for classification purpose in this proposed approach. The researchers implemented a hybrid feature extraction and selection method using Sequential Floating Forward Search (SFFS) where features are extracted from sensor signal using statistical formula to overcome the phenomenon of ‘curse of dimensionality’ [32]. They only rely upon some fixed statistical features whereas there exist many statistical features which they were totally overlooked. In one study [33], a dimensionality reduction-based scheme with feature extraction has been implemented using PCA but it lacks to produce the desired baseline. In 2015, Xizhe Yin et al. designed and tested four machine learning-based techniques namely J48, Support Vector Machine, Naive Bayes, and Multilayer Perceptron. The purpose was to detect five activities where data was composed from 3-axial accelerometer, 3-axial linear accelerometer, gyroscope in different orientation. The whole process had 4 subsections. First, placement of smartphone the in human body, second, data accumulation, third, feature extraction from raw data and then classification. Decision tree based J48 algorithm generated output with an average accuracy of 96.8%. In addition, the accuracy of all other algorithms was over 99%. At the conclusion the experiment showed that J48 is more user friendly in terms of simplistic IF-THEN rubrics but the rest of the classifiers could produce contented outcomes [34]. The implemented model of Li Fang et al. used feature extraction as a preprocessing technique and Support Vector Machine (SVM), K-Nearest Neighbor and Logistic Regression as classifiers. The experiment got a usual accuracy 88.9% (SVM), 95.3% (KNN) and 83.9% (Logistic Regression). The study also engrossed on up and down from buses. For both actions, the study achieved an accuracy 89.5% for SVM, 96.8% for KNN, 89.7% for LR for up into bus, for down from bus 84.2% for SVM, 94.2% for KNN, 81.9% for LR [35]. Though, procedural machine-learning applications produce good performance but they depend on domain-based information.

To solve this problem, activity recognition is shifting towards the deep learning-based techniques [27,36]. A Convolutional Neural Network (CNN) can automatically extract feature which is proposed in [26]. This study thus uses deep learning-based shallow CNN methodology which is an unsupervised approach and extracts local and statistical features. This is capable of extracting universal characteristics of sensor data [22]. In another study [36], a 3-layer LSTM model achieves an accuracy of 97.4% which is based on layer wise LSTM and CNN models. In one study [37], a low-cost Logistic Model Tree (LMT) is proposed for identify the time segment data. In one study [38], a data driven approach has been proposed. In one study [39], Probabilistic Neural Network and Fuzzy Cluster algorithm is proposed for the incremental learning ability but it reduces the required accuracy. In one study [40], a special kind of deep neural network has been proposed which is a combination of convolutional layers and short-term memory (LSTM). With a few model parameters it can automatically extract features. In one study [41], backward locking phenomenon is reduced and it is found that layer wise CNN with local loss exhibits good outcomes. It uses few numbers of parameters. However, if it uses higher parameters then it might exhibit some discrepancy. Ran Zhu et al. implemented a model that uses three different data sources i.e., data gathered from accelerometer, gyroscope and magnetometer. There were a total of 100 subjects who generated the sensor data. The machine learning-based approach was implemented on preprocessed data particularly Convolutional Neural Network (CNN). It is found that this study gained a rationally satisfactory result of 95.62% accuracy. Also, 96.29% accuracy was gained by the same research by using novel ensembles of CNN [42]. In one study [24], the Stack AutoEncoder (SAE) and heuristic optimization algorithm-based artificial bee colony (ABC) were proposed. In one study [43], series data are converted into images using computer vision methodology and employed a deep learning approach. To overcome the imbalance distribution of labeled data in semi-supervised learning the researchers proposed a semi-supervised deep Recurrent-Convolutional-Attention model [25]. In addition, in a study by [44], it was interestingly found that an unsupervised learning technique is not capable to handle such situation. Mohammed et al. collected the activity data from human wearable sensors. Sensors were equipped with accelerometer and gyroscope to produce three axial raw data. Then they preprocessed the data with Kernel Principal Component Analysis and Linear Discriminant Analysis to intensification the model robustness so that the existing features are good enough for accurate classification. At the end, preprocessed data was trained with a Deep Belief Network (DBN). Also, they associated the outcomes with other recognition models such as conventional SVM and ANN and claimed to reach an accuracy of 95.85% [45].

In a current scenario, insignificant research on the transferring of deep learning model has been carried on in this area. Strength and flexibility of activity recognition models can be more smartly understood by transfer learning approaches with the help of previous tasks. Evocative knowledge transmission depends on the relationship on a source and a target domain which can successfully accomplished by transfer learning [46]. In one study [47] on deep transfer learning between subjects and a target of unlabeled data, the researchers used deep transfer learning. Thus, a CNN model is deployed for calculating the distance between inner and inter class. However, from the experiment, it shows that knowledge transfer is hampered by large inner-class distance and small inter-class distance which is ultimately solved by a combination of the MMD method with central loss as this technique is able to lessening the inner-class detachment and rise method recital.

Deep learning methodology not only produces a balanced generalization but also totally eliminate the need for manual feature engineering with a high accuracy. However, deep learning-based techniques demand a high computational cost and a large number of annotated samples. Considering every issues in mind, this study implements a low-cost automatic optimal feature extraction method for HAR.

## 3. Methodology

**Figure 1:**
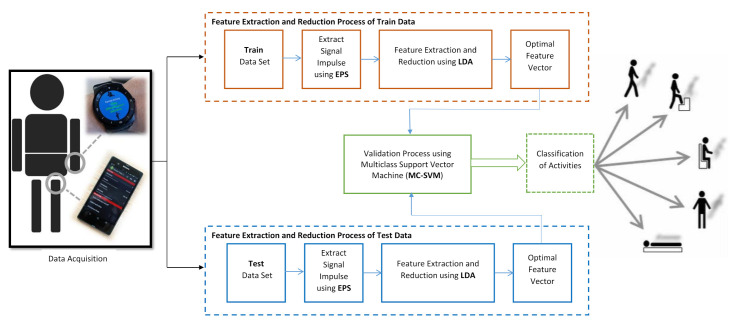
Working flow-diagram of the proposed model.

Accelerometer and gyroscope data are the main source of signal for identification of human activity. The overall process is depicted with the help of block diagram which is shown in Figure 1. The total process is divided into three subsections: (1) Data Gather (2) Good Feature Extraction and (3) Classification.

### 3.1. Proposed Feature Extraction and Reduction

**Figure 2:**
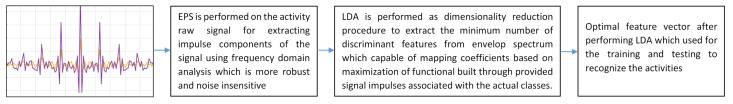
Working procedure of the feature extraction process.

Embedded noise is a natural phenomenon in any signal. Thus, to get rid of unwanted data from signal, many feature extraction techniques are available for correct classification. This research is emphasized on enveloped power spectrum (EPS). EPS extracts impulse from raw data. After completion this process, vocabulary extraction and dimensionality reduction are the next tasks. These tasks are performed with Linear Discriminant Analysis (LDA) from the impulse i.e., signal spectrums. Figure 2 shows the working procedure of the feature extraction process.

#### 3.1.1. Enveloped Power Spectrum (EPS)

EPS can be classified as an imperative use of Digital Signal Processing (DSP). For frequency domain analysis it is more robust and noise insensitive. EPS estimates the intermittent and irregular signals. EPS can check the irregularities in the periodograms of the time series signal [48]; particularly signals that are generated from different machines. Sensors (for example: accelerometer and gyroscope) are a good example for such signals. This powerful EPS technique is measured from sensor signals with the help of Fast Fourier Transform (FFT). In here, periodogram function assesses the power spectrum and can be defined over the N-point sequence y[n].

(1) $$I_{N}\left(\omega \right)=\frac{1}{N}{\left|Y\left(\omega \right)\right|}^{2}$$

where

(2) $$Y\left(\omega \right)=\sum_{n=0}^{N-1}y\left[n\right]e^{-jwnT}$$

Y(ω) is the discrete-time Fourier change of y[n].

(3) $$R\left(n\right)=\frac{1}{N}\sum_{k=0}^{N-1}y\left[n+k\right]\bar{y}\left[k\right]\;\;for\;\;\left|n\right|<=N-1$$

or,

(4) R(n)=0elsewhere

**Figure 3:**
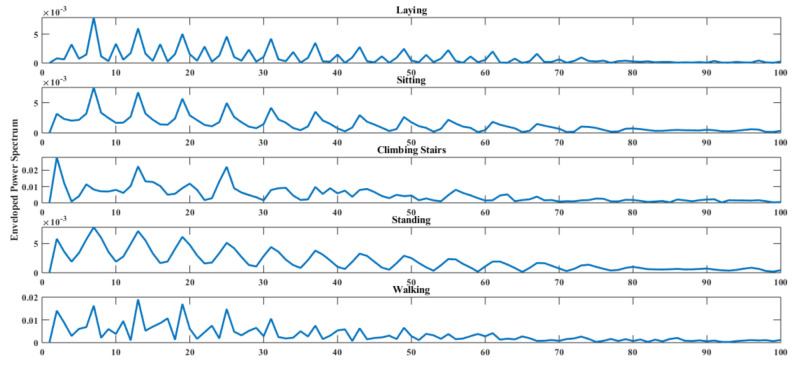
Enveloped power spectrum of sample signals of five activities.

From experience, general tendency is that the converse change in periodogram exhibits by the sample auto relationship function R(n). Figure 3 shown the power spectrum of each activity signal.

#### 3.1.2. Linear Discriminant Analysis (LDA)

Additionally, maximum separation of class is required for better classification. LDA explores the directions for maximum separation. Among the many LDAs, Fisher’s Linear Discriminant Analysis provides rational separation between various classes of data that leads to precise data classification [49]. LDA’s class covariance matrix can be defined by,

(5) $$S_{w}=\sum_{k=1}^{k}S_{k}$$

where

(6) $$S_{k}=\sum_{n\epsilon c_{k}}\left(x_{n}-m_{k}\right){\left(x_{n}-m_{k}\right)}^{T}$$

and

(7) $$m_{k}=\frac{1}{N_{k}}\sum_{n\epsilon c_{k}}x_{n}$$

Here, in class $C_{k}$, $N_{k}$ is the count of total patterns. In addition $X_{n}$ is the DWT coefficient of $n^{\mathrm{th}}$ pattern where *k* is the full class numbers. The covariance matrix between the class is characterized as,

(8) $$S_{B}=\sum_{k=1}^{k}N_{k}\left(m_{k}-m\right){\left(m_{k}-m\right)}^{T}$$

where

(9) $$m=\frac{1}{N}\sum_{n=1}^{N}x_{n}=\frac{1}{N}\sum_{k=1}^{K}N_{k}m_{k}$$

is the global mean of the data. The total covariance matrix is defined as,

(10) $$S_{T}=S_{B}+S_{W}$$

To end, projection matrix is calculated by,

(11) $$W=arg_{W}max\left\{{\left(WS_{W}W^{T}\right)}^{-1}\left(WS_{B}W^{T}\right)\right\}$$

The LDA coefficients were obtained from the projection matrix as,

(12) $$y=W^{T}x$$

Here, *x* vector is the DWT coefficient (the low and high frequency components, of the input signal at various levels) and *y* vector is the LDA coefficients.

### 3.2. Classification

At the final stage of our proposed model, a non-linear MCSVM has been implemented to classify the individual activity. In general, SVM is a very good supervised machine algorithm that for both classification and regression. It is widely used as a binary classifier to analyze and recognize the patterns [2].

The main idea of SVM is to use a hyper-plane for binary classification. However, in many cases, dataset is nonlinear and therefore cannot be classified with a single hyper-plane. In these cases, a kernel function might be a perfect choice. It has the ability to classify nonlinear data. There are few versions of nonlinear kernel function such as: polynomial functions, Gaussian radial basis function and the hyperbolic tangent. In this experiment, widely used Gaussian radial basis kernel function has been implemented. This function can be defined as

(13) $$k\left(xv_{a},xv_{b}\right)=\exp\left(\frac{||xv_{a},xv_{b}{\left|\right|}^{2}}{2\gamma^{2}}\right)$$

The function k progressions separated binary inputs $xv_{a}$, $xv_{b}$ as parameters. The feature vectors or input parameters are calculated with another autonomous variable to speculate the width of active basis function kernel, also designated as γ.

**Figure 4:**
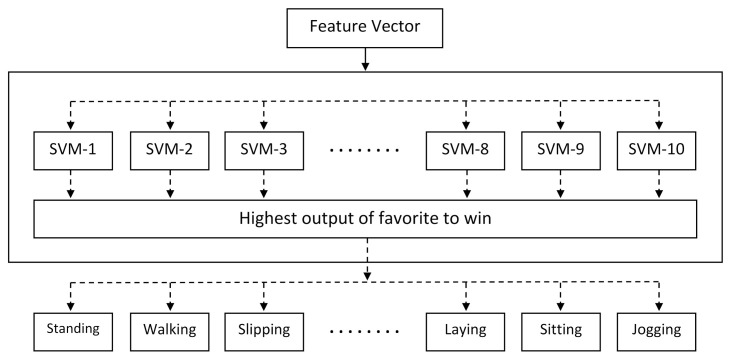
Architecture of OAA SVM classifier for activity recognition.

There are few SVM implementations such as follows: one-against-all (OAA), one-against-one (OAO), and one-acyclic-graph (OAG). In this experiment, least complicated multi-class nonlinear classification method (OAA) has been used. The OAA-MCSVM has *N* SVMs and can work in parallel, as shown in Figure 4. Every SVM distinguishes one class from other classes and lastly a choice can be made by choosing the SVM which comprises the major output value.

## 4. Experiment

### 4.1. Data Description

The UCI-HAR dataset [17] and DU-MD dataset [14] are used to evaluate the performance of the proposed model by conducting extensive experiments. Both datasets are publicly available for research purposes.

#### 4.1.1. UCI-HAR Dataset

**Figure 5:**
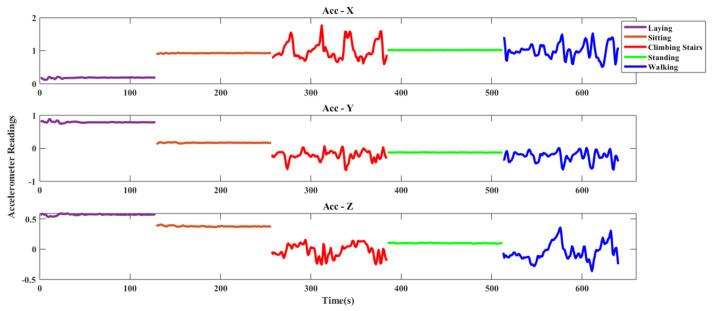
Triaxial accelerometer data of UCI-HAR dataset.

**Figure 6:**
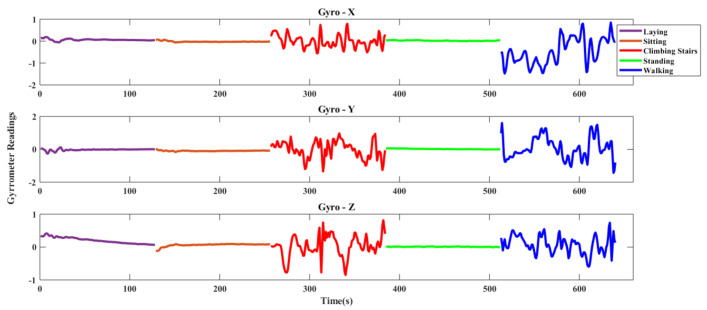
Triaxial Gyroscope data of UCI-HAR dataset.

In this dataset, the researchers observed five daily activities of thirty volunteers. The age range of the subjects varies from nineteen to forty-eight (19–48). A waist-mounted smartphone generated the desired data of five popular human activities. These were: walking, laying, sitting, standing and climbing stairs (both upstairs and downstairs). For data generation, smartphone used two sensors: accelerometer and gyroscope. Accelerometer calculated the triaxial linear acceleration and gyroscope calculated the triaxial angular velocity with a constant rate of 50 Hz. The activities were marked manually with the help of recorded video. The full dataset was splitted into two subsets: 70% data were selected for generating training data and rest of the data were chosen for testing. Also, sensor signals were pre-processed and low-pass filters were used for sampling in fixed-width sliding windows of 2.56 s with a 50% overlap where window width is 128. Table 1 shows the class-wise data distribution of the UCI-HAR dataset. Figure 5 and Figure 6 show the triaxial accelerometer and triaxial gyroscope data respectively of a sample activity signal of UCI-HAR dataset.

#### 4.1.2. DU-MD Dataset

**Figure 7:**
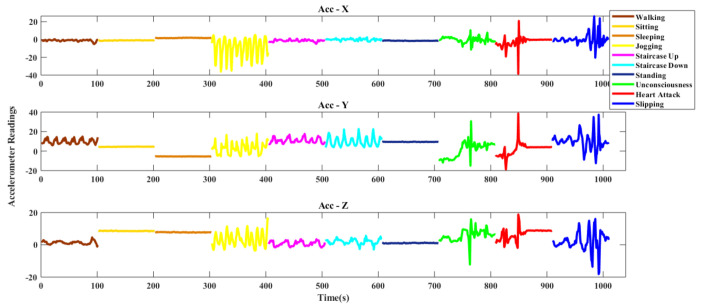
Triaxial accelerometer data of DU-MD dataset.

This is also a public dataset. In this dataset, the researchers observed several activities. There were seven basics (Walking, Sitting, Sleeping, Jogging, Staircase Up, Staircase Down, Standing) and three falls (Falling Unconsciousness, Falling Heart Attack, Falling Slipping) activities of thirty-four (34) persons. Each activity has by ten signals for each person with 101 samples. Figure 7 shows the triaxial accelerometer data of DU-MD dataset.

### 4.2. Experimental Setup and Performance Measurement Criteria

Every experiment is implemented on a laptop computer Intel(R) Core(TM) i5-6200U 2.30 GHz and 8 GB RAM with operating system windows (x64) version 10, and using MATLAB programming language tool. To evaluate the performance of the proposed model, four evaluation metrics are used and these four evaluation metrics are computed as follows:

(14) $$Accuracy=\frac{T_{p}+T_{n}}{T_{p}+F_{p}+F_{n}+T_{n}}$$

(15) $$Precision\left(P\right)=\frac{T_{p}}{T_{p}+F_{p}}$$

(16) $$Recall\left(R\right)=\frac{T_{p}}{T_{p}+F_{n}}$$

(17) $$F_{1}\;Score=\frac{2\times (P\times R)}{P+R}$$

A signal belonging to one class may be misclassified as belonging to another, creating a false positive recognition ($F_{p}$) of that class, while a signal belonging to another class may be misclassified as belonging to that class, creating a false negative ($F_{n}$) recognition of that class. When the class of a considered signal is accurately predicted, the recognition is defined as a true positive ($T_{p}$) for the considered class and as a true negative ($T_{n}$) for all other classes.

### 4.3. Feature Extraction and Reduction Analysis

**Figure 8:**
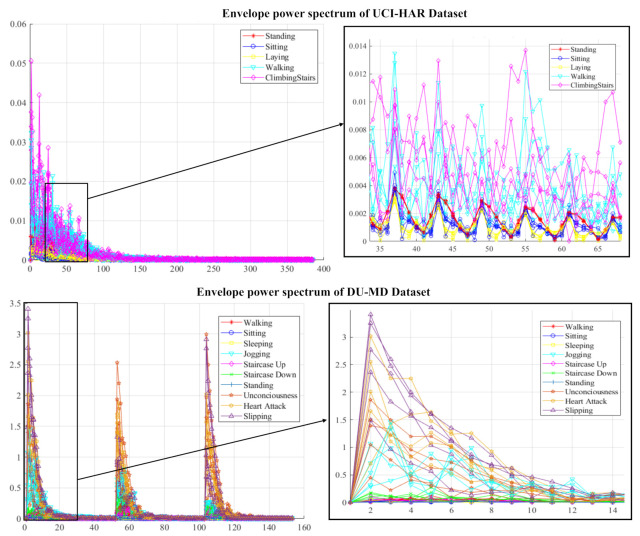
Signal impulse of human activities.

**Figure 9:**
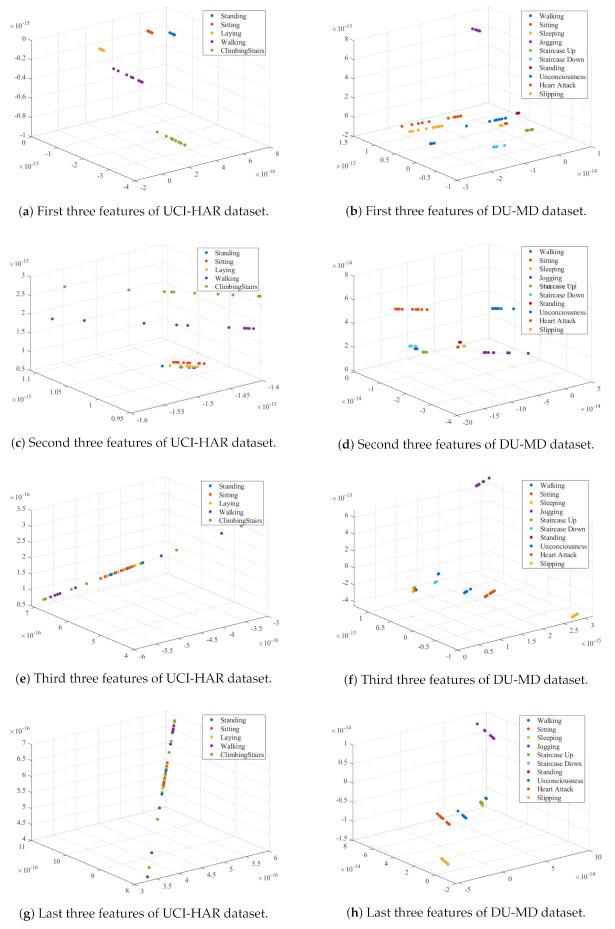
3-D LDA features plot of both datasets.

At first, signal impulses are extracted from each activity raw signal using the enveloped power spectrum (EPS). The Enveloped Power Spectrum (EPS) is used for extracting impulse components of the signal using frequency domain analysis which is more robust and noise insensitive. After applying the EPS, 153 and 385 signal spectrums are obtained from 303 and 768 samples of a signal for the DU-MD and UCI-HAR datasets respectively. Figure 8 shows the signal impulses of human activities. The Linear Discriminant Analysis (LDA) is used as dimensionality reduction procedure to extract the minimum number of discriminant features from envelop spectrum. LDA capable of mapping coefficients based on maximization of functional built through provided signal impulses associated with the actual classes. After performing LDA, the feature vectors have become 49 and 123 for each activity signal. In the first order dimension LDA provides discriminant features and are class-wise separable but in the higher order dimension LDA features are not that much discriminative. Figure 9 shows the class-wise features in six subsections for visual representation. Figure 9a,b shows the LDA implanted first three features of each class of the UCI-HAR and DU-MD datasets. It is clearly visible that the first three features of each class are fully separable on a three-dimensional cartesian coordinate system plane. The second three features of each class have a minor over-fitting but still separable for the both datasets which are shown in Figure 9c,d respectively. On the other hand, Figure 9e–h state that these features of for the both datasets are over-fitted between classes and thus those are not separable.

### 4.4. Result Analysis

**Figure 10:**
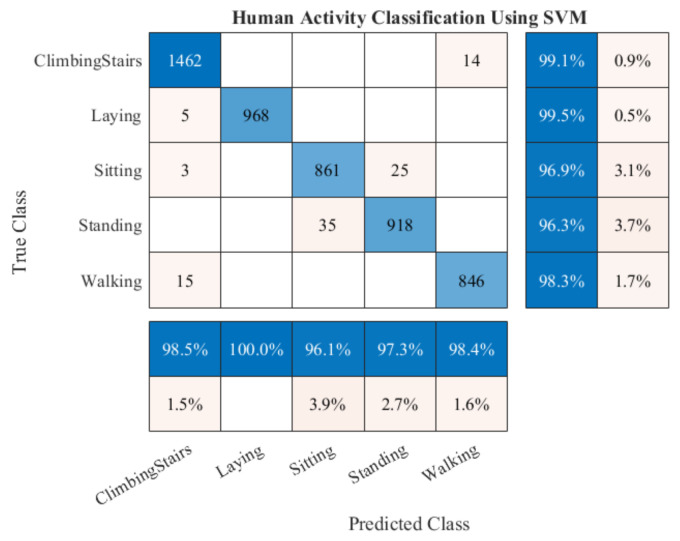
Confusion matrix of the proposed model on UCI-HAR dataset for the five best features.

**Figure 11:**
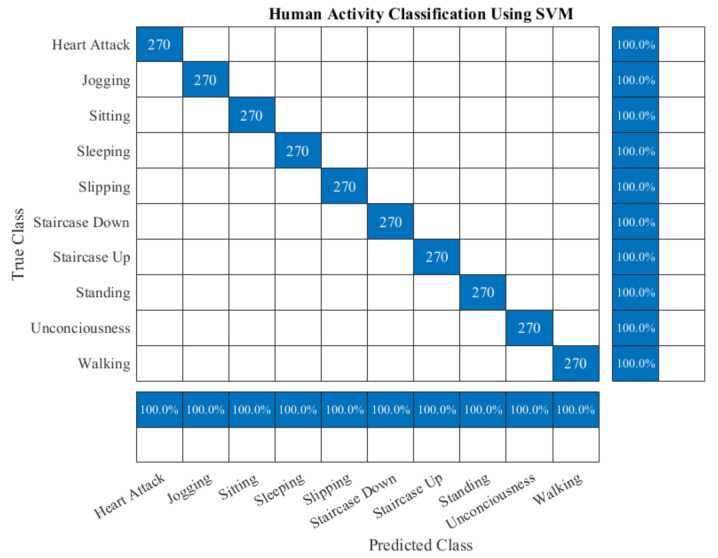
Confusion matrix of the proposed model on DU-MD dataset for the five best features.

**Figure 12:**
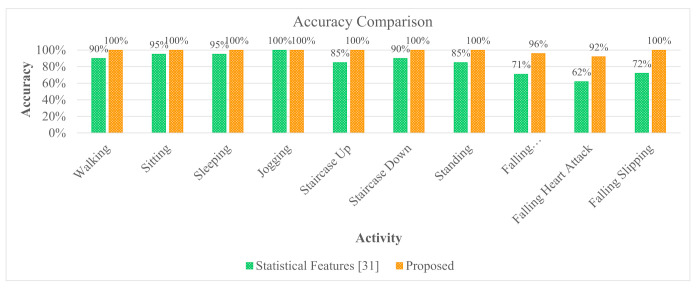
Class-wise accuracy comparison of the proposed model with other state-of-the-art models on the DU-MD dataset.

In this experiment, EPS and LDA based feature extraction and reduction model are performed on the raw activity signals for getting the discriminant features. This model uses the two well-known publicly available datasets namely DU-MD and UCI-HAR datasets. These two datasets are branched into two subsets for the training and the testing. For the training purpose, 50% data is used and rest of the data is used for testing. After feature extraction and dimensionality reduction, feature vectors are trained using a conventional and very well-known supervised classifier, specifically, Support Vector Machine (SVM). Though SVM is binary in nature still this classifier can also be used as multiclass classifier efficiently. After completing the training, the performance of the implemented model evaluated by the test data. Classifier is trained and tested for considering different number of features for the both datasets which is shown in Table 2. This experiment illustrates the classification performance increases by using the more features but there exists an optimal number of features where maximum performance is gained. After that, it exhibits an inverse relation between the increasing number of features and classification performance. Table 2 shows the performance of the classifier using different number of features. It is found that the best performance of this experiment is obtained from top five features for the both datasets. The classifier shows 98.67% accuracy and 98.71% F1 score on the UCI-HAR dataset. Similarly, 100% accuracy and 100% F1 score are obtained from the same model on the DU-MD dataset. Figure 10 and Figure 11 show the confusion matrix to visualize the performance of the implemented model in a contingency table on the both datasets respectively. Next, we have applied 3-fold cross-validation on the both datasets to check the robustness of the proposed model. First 2-fold used for training and rest of the fold used for testing purpose. From this experiment, its found that the performance of the proposed model has increased due to the number of training set has increased which is shown in Table 3. Figure 12 shows the class-wise accuracy comparison of the proposed model with other state-of-the-art models on the DU-MD dataset. The class-wise accuracy comparison of the proposed model with other state-of-the-art models on the UCI-HAR dataset is shown in Table 4. The performance comparison of the implemented model with the other state-of-the-art methods are shown in Table 5 and Table 6 as well. From Table 5 and Table 6, it is clear that the implemented model gives superior performance than the other state-of-the-art methods in terms of accuracy and cost.

## 5. Conclusions

This paper presents an effective human activity recognition system. In this model, the EPS- and LDA-based feature extraction and reduction model have been introduce for smartphone sensor data. To increase the classification performance, quality feature extraction is a prime target from raw sensing data because of unwanted noise. The main advantage of the proposed EPS- and LDA-based feature extraction and reduction model can reduce the noise and extract the quality features from accelerometer data and gyroscope data. To judge the performance of the proposed system, a supervised classification model is incorporated in this study. To validate the system, the UCI-HAR and the DU-MD datasets are used. The experimental results show superior performance compared with other feature extraction methods, and with deep learning-based state-of-the-art methods in terms of performance and cost. The conclusions made with this research help to encourage future work and introduce a new project on the activity recognition systems using wearable sensors. In the future, new adaptations will be introduced on the proposed feature extraction and reduction model such as preprocessing, applying filters, and advance feature selection methods that can be evaluated. Besides, we will try to apply unsupervised techniques to make the HAR systems more robust.

## Figures and Tables

**Table 1:** Class wise data description of UCI-HAR dataset.

Activity	Number of Signal	Each Signal Dimension
Laying	1944	768
Sitting	1777	768
Standing	1906	768
Walking	1722	768
ClimbingStairs	2950	768

**Table 2:** Feature based performance (%) of the proposed model on both datasets.

No. of Features	UCI-HAR Dataset				DU-MD Dataset			
	Accuracy	Precision	Recall	F1 Score	Accuracy	Precision	Recall	F1 Score
**5**	**98.67**	**98.67**	**98.75**	**98.71**	**100**	**100**	**100**	**100**
10	93.33	93.33	93.89	93.61	99.33	99.33	99.37	99.35
15	89.33	89.33	90.41	89.87	98.00	98.00	98.33	98.17
20	85.33	85.33	91.54	88.33	94.00	94.00	95.00	94.50
25	84.00	84.00	84.91	84.45	94.00	94.00	95.00	94.50
30	82.67	82.67	83.73	83.19	91.33	91.33	93.51	92.41
35	78.67	78.67	84.07	81.28	89.33	89.33	92.08	90.69
40	77.33	77.33	87.65	82.17	82.00	82.00	87.28	84.56
All	42.67	42.67	45.17	43.88	33.33	33.33	41.40	36.93

**Table 3:** Feature based performance (%) of the proposed model performing 3-fold Cross-Validation on both datasets.

No. of Features	UCI-HAR Dataset				DU-MD Dataset			
	Accuracy	Precision	Recall	F1 Score	Accuracy	Precision	Recall	F1 Score
**5**	**99.73**	**99.69**	**99.77**	**99.73**	**100**	**100**	**100**	**100**
10	95.00	95.67	95.00	95.33	100	100	100	100
15	91.00	91.18	91.00	91.09	100	100	100	100
20	87.33	87.63	87.33	87.48	98.00	98.33	98.00	98.17
25	86.00	87.14	86.00	86.57	96.00	97.14	96.00	96.57
30	83.73	83.77	83.69	83.73	93.00	95.38	94.00	94.69
35	80.00	80.39	81.00	80.83	90.00	92.47	90.00	91.22
40	79.00	79.14	79.00	79.07	82.00	88.13	82.00	84.95
All	46.33	47.29	48.00	47.83	36.00	36.25	36.00	36.12

**Table 4:** Class-wise accuracy (%) comparison of the proposed model with other state-of-the-art models on the UCI-HAR dataset.

Activity	Feature Level Fusion+SVM [5]	CNN [22]	Hybrid Feature Selection [32]	Proposed Model
Laying	100	99.40	99.26	100
Sitting	98.90	90.04	97.76	**100**
Standing	98.14	98.20	97.18	**100**
Walking	99.88	99.40	98.99	93.33
ClimbingStairs	99.96	98.81	97.24	**100**

**Table 5:** Performance comparison of the proposed model with other state-of-the-art methods on the DU-MD dataset.

Authors	Methods	No. of Features	Accuracy (%)
Saha et al. [31]	DWT+SF+SVM	39	90.50
	DWT+SF+EoC		93.00
**Proposed Model**	**EPS+LDA+MCSVM**	**5**	**100**

**Table 6:** Performance comparison of the proposed model with other sate-of-the-art models on the UCI-HAR dataset.

Author	Methods	Accuracy (%)	Each Sample Classification Time
Chen et al. [25]	Recurrent Convolutional Attention	81.32	21.538 ms ∼ 57.253 ms
Ignatov et al. [22]	CNN	97.63	33.286 ms ∼ 35.714 ms
Teng et al. [41]	CNN+Baseline (global loss)	96.20	24.462 ms ∼ 26.055 ms
	CNN+pred (local loss)	95.42	
	CNN+sim (local loss)	96.16	
	CNN+predsim (local loss)	96.98	
Tufek et al. [36]	2D CNN	85.20	331.27 ms ∼ 338.03 ms
	1D CNN+LSTM	88.50	
	2 layer LSTM	93.70	
	3 layer LSTM	97.40	
Jain et al. [5]	Score-level fusion+KNN	84.02	–
	Feature-level fusion+KNN	91.75	
	Score-level fusion+SVM	96.44	
	Feature-level fusion+SVM	97.12	
Xia et al. [40]	LSTM-CNN	95.78	340.62 ms ∼ 349.80 ms
**Proposed Model**	**EPS+LDA+MCSVM**	**98.67**	**0.0500 ms ∼ 0.1235 ms**
